# Supplementary material for: A course-based undergraduate research experience examining neurodegeneration in Drosophila melanogaster teaches students to think, communicate, and perform like scientists
Source: PLoS One. 2020 Apr 13;15(4):e0230912. doi: 10.1371/journal.pone.0230912 (PMC7153876; doi:10.1371/journal.pone.0230912)
Supplement: S1 File — Adapted from [28, 29]. (DOCX) [file pone.0230912.s002.docx]

**CLIMBING ASSAY PROTOCOL**

1. Gather fresh food vials that all have approximately the same level of food at the bottom.
2. Over light CO_2_ anesthesia, gather a group of approximately 10 male flies and place them in a food vial.
3. Tape an empty (no food) vial on top of the food vial with the flies and wait at least 1 hour for the flies recover from the CO_2_ (best done in morning or afternoon of testing day).
4. Repeat for all remaining groups of flies to be tested (replicate groups of the same genotype, as well as control genotypes).
5. (While a partner videotapes on a smartphone,) tap flies down to the bottom of the food vial on a mousepad mat and allow them to climb for 20 seconds.
6. Repeat this four more times with same group of flies (for a total of 5 climbing trials per group).
7. Repeat with remaining groups of flies.

|  | Genotype | Time put in vial | Time tested | Trial 1 | Trial 2 | Trial 3 | Trial 4 | Trial 5 |
| --- | --- | --- | --- | --- | --- | --- | --- | --- |
| Group 1: |  |  |  |  |  |  |  |  |
| Group 2: |  |  |  |  |  |  |  |  |
| Group 3: |  |  |  |  |  |  |  |  |
|  |  |  |  |  |  |  |  |  |
|  |  |  |  |  |  |  |  |  |
|  |  |  |  |  |  |  |  |  |
|  |  |  |  |  |  |  |  |  |
|  |  |  |  |  |  |  |  |  |

Analysis/Quantification of Climbing Assay

Rename and save video file with the following convention:

Year_month_day_timetested_initials_genotypeshorthand_group#

For example:

20181022_18:36_BD_wtctl_group1

Transfer video file to lab’s common external hard drive

Review the video file carefully to count how many flies climb out of the bottom vial and into the top empty vial during each trial.

Over the 5 trials, each time a fly climbs out of the food vial earns 1 point. The points from all 5 trials are summed together, then divided by the total number of flies in the group (should be 10 flies) to yield the Climbing Index.

For example:

| Trial 1 | Trial 2 | Trial 3 | Trial 4 | Trial 5 | Sum | Climbing Index |
| --- | --- | --- | --- | --- | --- | --- |
| 9 | 10 | 8 | 9 | 10 | 46 | 4.6 |

At least four groups should be tested for each condition/genotype (can be gathered over multiple days) and the Climbing Indices are averaged, reported with standard deviation.
