# Supplementary material for: A course-based undergraduate research experience examining neurodegeneration in Drosophila melanogaster teaches students to think, communicate, and perform like scientists
Source: PLoS One. 2020 Apr 13;15(4):e0230912. doi: 10.1371/journal.pone.0230912 (PMC7153876; doi:10.1371/journal.pone.0230912)
Supplement: S2 File — Adapted from Sarah Petersen, Kenyon College, personal communication to R.D. (DOCX) [file pone.0230912.s003.docx]

**GUIDED READING WORKSHEET**

| **BACKGROUND AND OVERVIEW** | |
| --- | --- |
| **Article Title/Citation**  (and url, if web accessed) |  |
| **Study objectives/purpose**  (and research hypothesis, if applicable)  *What BIG question is this article trying to answer?* |  |
| **Brief background** (why issue is important, summary of previous literature)  *Why did the authors do this study?* |  |
| **Key Words** (list terms used repeatedly, with definitions in your own words) |  |
| **Funding sources** |  |
| **METHODS** | |
| **Study design and methodology** (type of study, controls, etc.)  *How did the authors do this study?* |  |
| **Statistical analyses** |  |
| **RESULTS** | |
| **List of results by each Figure/Table**  *What specific question is each figure trying to answer?* |  |
| **Brief summary of results as a whole**  *What data/results emerged from the study?*  *Do they answer the BIG question posed initially?* |  |
| **DISCUSSION & CONCLUSIONS** | |
| **Brief summary of authors’ main discussion points and conclusions**  *What do the authors think the results mean?* |  |
| **Study strengths, significance and impact**  *What is the significance of these findings?* |  |
| **Study limitations, weaknesses, potential for bias, etc.**  *Do the authors identify any weaknesses? Do you see any that the author missed?* |  |
| **Student conclusions and recommendations**  *Do you agree with the authors? What experiment would you do next?* |  |
