# Supplementary material for: A course-based undergraduate research experience examining neurodegeneration in Drosophila melanogaster teaches students to think, communicate, and perform like scientists
Source: PLoS One. 2020 Apr 13;15(4):e0230912. doi: 10.1371/journal.pone.0230912 (PMC7153876; doi:10.1371/journal.pone.0230912)
Supplement: S3 File — Adapted from [48]. (DOCX) [file pone.0230912.s004.docx]

**FIGURE/TABLE ANALYSIS WORKSHEET**

*Fill out one worksheet for each figure or table.*

Figure or Table Number: ___________

1. “Official” title for this figure or table (from the caption):

2. My (simplified, decoded, in regular language) title for this figure or table:

3. The specific hypothesis being tested, or specific question being asked in the

experiment represented here is:

Analysis:

4. For Descriptive figures/tables:

If we compare panels/columns ____ and ____, we learn about: (*repeat this as needed to describe all relevant comparisons*)

**OR**

4. For Experimental figures/tables:

-The **controls** in this experiment are:

and they are represented (in which part of the graph or in what figure panels):

-The **experimentals** are:

and they are represented:

-The **method** used in this figure is (*brief summary of key points in your own words, not as detailed as described in the methods section*):

We need to compare the controls represented in ______ with the experimentals in_______ to find out: (*repeat this as needed to describe all relevant comparisons*)

5. Overall, what we learn from the figure is:

6. The following issues are ones of concern for me (*These can be things that you*

*don’t understand, criticisms of methods/analysis, questions for the authors, anything else*

*that comes to mind*):
