# Supplementary material for: A course-based undergraduate research experience examining neurodegeneration in Drosophila melanogaster teaches students to think, communicate, and perform like scientists
Source: PLoS One. 2020 Apr 13;15(4):e0230912. doi: 10.1371/journal.pone.0230912 (PMC7153876; doi:10.1371/journal.pone.0230912)
Supplement: S4 File — (DOCX) [file pone.0230912.s005.docx]

**JOURNAL CLUB INSTRUCTOR GUIDELINES**

Journal Club discussion is conducted as a “Jigsaw” activity. First, small groups are formed initially in which everyone in the group discusses the same figure(s). Then groups are broken up and new groups are formed that contain one “representative” from each of the prior groups (so that each student in the 2^nd^ set of groups is responsible for explaining their figure(s) to the other students in the group).

**1. Introduce the activity to students** (describe flow of discussion, so everyone knows they’ll be responsible for explaining the material)

**2. Form 3 groups of 5 ppl: (10-15min total)**

*Numbers of groups and students in each group will vary depending on class size and number of figures in paper*

-count off to 3: 1,2,3

-pass out handout of discussion questions for each group

Fig 2,3, table1

Fig 4,5,6

Fig 7,8,9

-circulate, spend 2-3 min sitting in on each group

**3. Mix groups—teach each other as representatives (10-15 min)**

5 groups of 3 ppl (1 from each group)

-count off in each group to 5: 1,2,3,4,5

-instruct students to explain what they learned from their assigned section of the article to the group

-circulate, spend 2-3 min sitting in on each group

**4. Whole class discussion (20-30 min)**
