# Supplementary material for: A course-based undergraduate research experience examining neurodegeneration in Drosophila melanogaster teaches students to think, communicate, and perform like scientists
Source: PLoS One. 2020 Apr 13;15(4):e0230912. doi: 10.1371/journal.pone.0230912 (PMC7153876; doi:10.1371/journal.pone.0230912)
Supplement: S5 File — In reference to [49]. (DOCX) [file pone.0230912.s006.docx]

**JOURNAL CLUB DISCUSSION QUESTIONS**

Figures 2,3, table 1

-State main “take-home” point from each figure/table.

-What are some questions you still have (things that are unclear)?

-What more information would you like from the authors? Additional data? Different comparisons?

-What is the purpose of each of the three vectors illustrated in Figure 2A?

-How did the authors generate and screen multiple lines of pGawB with distinct expression patterns? What is the advantage of this over subcloning of a defined promoter region?

-What proof of principle is Figure 2B meant to demonstrate?

-Is pGawB inserted at the *hairy* locus in line IJ3?

(a) What two lines of evidence suggest that it is?

(b) How could we use a more modern technique to confirm the location of this insertion?

-What observation concerning IJ3-GAL4 > UAS-lacZ suggests that one should use caution in ascribing the expression pattern of a GAL4-driven reporter gene to the endogenous gene regulated by that particular enhancer?

Figures 4,5,6

-State main “take-home” point from each figure

-What are some questions you still have (things that are unclear)?

-What more information would you like from the authors? Additional data? Different comparisons?

-Is pGawB inserted at the *hairy* locus in line IJ3?

(a) What two lines of evidence suggest that it is?

(b) How could we use a more modern technique to confirm the location of this insertion?

-What observation concerning IJ3-GAL4 > UAS-lacZ suggests that one should use caution in ascribing the expression pattern of a GAL4-driven reporter gene to the endogenous gene regulated by that particular enhancer?

-in Figure 5, what point about the Gal4 system were the authors trying to make by also showing the effect of expressing *even-skipped* with *paired-Gal4*, after *hairy-Gal4*?

-How do the authors propose that misexpression of *even-skipped* results in the phenotype observed in Fig. 5? And what does that suggest about *even-skipped*’s normal function?

Figures 7,8,9

-State main “take-home” point from each figure

-What are some questions you still have (things that are unclear)?

-What more information would you like from the authors? Additional data? Different comparisons?

-Why was it significant that the *UAS-dRas2^Val4^* transgenic flies were viable and didn’t show any phenotypes?

-Were there other effects of Gal4-driven expression of *UAS-dRas2^Val4^* besides the eye and wing phenotypes shown in Figs 7 and 8?

-Why were more Gal4 lines characterized in Fig 9 than shown in Figs 7 and 8?

-What do the authors suggest can be done next using this system to study other genes in the Ras pathway?
