# Supplementary material for: A course-based undergraduate research experience examining neurodegeneration in Drosophila melanogaster teaches students to think, communicate, and perform like scientists
Source: PLoS One. 2020 Apr 13;15(4):e0230912. doi: 10.1371/journal.pone.0230912 (PMC7153876; doi:10.1371/journal.pone.0230912)
Supplement: S6 File — (DOCX) [file pone.0230912.s007.docx]

**GROUP PRESENTATION GUIDELINES**

Each group will deliver a 15-20 minute presentation, with an additional 5 minutes for audience questions. Your group will present on either: _____ or _____ (we will assign presentation dates through random drawing, but you are allowed to trade with other groups if they are willing).

All students will fill out peer evaluation forms to give feedback to each group on their presentation. Your attendance is required at all presentations.

Content:

*Background**

-Why is this general topic of research interesting or worth studying (make your audience care)?

-Include preliminary data that provides context and foundation for your results

-What is the central question you’re asking in this research project?

-Why did you set out to do the experiment that you did?

-Think of an inverted pyramid or funnel: start out broad and gradually get more specific until your reach your research question of interest

*Method**

-Why did you choose the methods you did?

-Brief, conceptual overview, don’t get bogged down in details

-*Drosophila* genetics, Gal4>RNAi, climbing assay

*Results*

-Can include qualitative observations (in list format)

-Include graphical representation of your climbing data (report statistics)

-Walk the audience through the first type of any graph (i.e. “on the x-axis you see..., on the y-axis...”)

*Conclusions*

-Summarize experimental findings

-What do you think your results mean?

-What caveats are there about your data (mistakes, inconsistencies, other variables) that could affect interpretation of the results?

*Future Directions*

-What additional experiments would you want to do to answer your specific research question?

-What other questions would be interesting to investigate next (beyond your specific objective for this semester, but related to the broader topic of Prof. Steinhauer’s ongoing research)?

*Some of this general information will be common to all groups, so where appropriate you should also try to provide information relevant to your driver/neuronal sub-type

*Background and Method information can be interspersed/overlap to some extent

Visual Presentation:

-Do not use too much text on slides

-Use consistent font and layout/design

-Consider use of a “home” slide with an outline of entire presentation that you can return to throughout presentation

Oral Presentation:

-Make eye contact with audience, speak clearly and confidently

-Do not simply read from your notes or slides

-Rehearse—especially transitions between slides and between partners

-Think about how you will divide presentation time so that each group member gets to demonstrate and practice presentation skills, while keeping overall presentation coherent

-Think about how you will respond to audience questions
