# Supplementary material for: A course-based undergraduate research experience examining neurodegeneration in Drosophila melanogaster teaches students to think, communicate, and perform like scientists
Source: PLoS One. 2020 Apr 13;15(4):e0230912. doi: 10.1371/journal.pone.0230912 (PMC7153876; doi:10.1371/journal.pone.0230912)
Supplement: S7 File — (DOCX) [file pone.0230912.s008.docx]

**GROUP PRESENTATIONS: GRADING RUBRIC**

Presenting Group: Total Points: ___/30 = ___%

| Organization | Background/Methods | Results | Conclusions/Future Directions | Visual presentation | Oral Presentation |
| --- | --- | --- | --- | --- | --- |
| 5  -appropriate time is spent on each section  -transitions between slides are smooth  -connections between major sections are logical  -order of information presented is logical  -information is presented in appropriate section | 5  -provides “why” the audience should be interested  -logically leads to the experiment being done  -neither excessive nor insufficient in detail  -central research question is clearly stated  -clear which methods were used and why | 5  -graphs are clear, easy to follow  -statistics are clearly stated  -qualitative observations are distinguishable from quantitative data  -results are set in context of broader research question  -complete/ comprehensive | 5  -communicates specific and logical conclusions from results  -addresses caveats  -interpretation of results and caveats is accurate  -follow-up experiments to address caveats are presented  -suggests appropriate future experiments | 5  -slides design/layout is visually appealing and not distracting  -consistent and appropriate font and size  -visual aids are effective  -graphs are clearly labeled  -not too much text on each slide | 5  -pacing is neither too fast nor too slow  -clear and confident speech  -clearly walks audience through graphs or diagrams  -transitions between group members are smooth and each group member is represented  -handles questions appropriately |
| 4 | 4 | 4 | 4 | 4 | 4 |
| 3 | 3 | 3 | 3 | 3 | 3 |
| 2 | 2 | 2 | 2 | 2 | 2 |
| 1 | 1 | 1 | 1 | 1 | 1 |
