# Supplementary material for: A course-based undergraduate research experience examining neurodegeneration in Drosophila melanogaster teaches students to think, communicate, and perform like scientists
Source: PLoS One. 2020 Apr 13;15(4):e0230912. doi: 10.1371/journal.pone.0230912 (PMC7153876; doi:10.1371/journal.pone.0230912)
Supplement: S8 File — (DOCX) [file pone.0230912.s009.docx]

**GROUP PRESENTATIONS: PEER FEEDBACK FORM**

Names of Presenting Group:

Scale

Strongly Disagree Disagree Neutral Agree Strongly Agree

1 2 3 4 5

Slides/Content

Was the presentation well organized (did it have a logical flow that you could easily follow)?

1 2 3 4 5

Did the slide layout and design contribute to the effectiveness of the presentation (with few or no distracting elements)?

1 2 3 4 5

Did the presentation clearly communicate why the experiments were done?

1 2 3 4 5

Did you understand the results the group obtained as they presented the data?

1 2 3 4 5

Describe one or more **strengths** of the slides:

Describe one or more ways to **improve** the slides:

Oral Presentation

Did the presenters speak clearly (could you easily understand them)?

1 2 3 4 5

Were they engaged with the audience (maintain good eye contact, etc.)?

1 2 3 4 5

Did they seem prepared/well-rehearsed (effective pacing, didn’t rely heavily on notes, concise delivery)?

1 2 3 4 5

Did they answer audience questions well?

1 2 3 4 5

Describe one or more **strengths** of the speakers:

Describe one or more ways the speakers could **improve** their oral presentation:
