# Supplementary material for: A course-based undergraduate research experience examining neurodegeneration in Drosophila melanogaster teaches students to think, communicate, and perform like scientists
Source: PLoS One. 2020 Apr 13;15(4):e0230912. doi: 10.1371/journal.pone.0230912 (PMC7153876; doi:10.1371/journal.pone.0230912)
Supplement: S9 File — Adapted from [33]. (DOCX) [file pone.0230912.s010.docx]

**LAB REPORT GUIDELINES**

For this lab, each student must submit a full lab report. Structure your lab report as a research paper, with the following sections: Abstract, Introduction, Methods, Results, Discussion, References. Use the following guidelines to help you prepare your lab report.

You should include a title at the beginning of your report. Take care that your grammar and spelling are accurate, and that your writing is logical and coherent. Do not use slang, contractions, or undefined abbreviations; always write scientific names correctly (i.e. *Homo sapiens*). A scientific paper should be written to conserve space, not take up as much room as possible. In a minimum amount of space you need to clearly describe what you did and explain its relevance to your reader. It is almost never appropriate to quote in a scientific paper, instead you should paraphrase (put the idea in your own words).

While you conducted the project in groups, the written paper you turn in should be in your own writing and should not copied from other group members or classmates. You are welcome to discuss and proofread each other’s papers, but your work should be your own.

Submit your lab report (as a Word doc) on Canvas by **­­­­­_________**.

For reports that are <24 hours late: -5% pts, 24-48 hours late: -10% pts, 48-72 hours late: -15% pts

**Reports more than 72 hours late will not be accepted without a doctor’s or dean’s note.**

Abstract

This is a very brief description of why you did it, what you did, how you did it, and what you found. It is best written last, using the most important 1-2 ideas from each section. It must clearly state your research question/objective, briefly describe the methods used and the results, and clearly state your conclusions. Your hypothesis or purpose and conclusions should be scientific, and should not refer to learning goals (“The purpose of this lab was to learn about *Drosophila* genetics,” is not acceptable).

Introduction

This section introduces the reader to the topic of your study and should entice the reader to keep reading. It is appropriate to include information about relevance to human health and disease. The introduction should contain some background information on the topic and a summary of the results and conclusions of other studies that have been conducted on the same subject. This background information should logically lead to your objective or research question, which should be put at the end of the introduction. Cite any literature you use to write this section (or any part of the report). See the CSE guide (at the end of this document) for directions on how to style these internal references. For preliminary data from Prof. Steinhauer that is unpublished, you can cite as “(Steinhauer, unpublished data).”

Methods

This section should be a detailed account of exactly what you did and what statistical tests you used, but it is written in third person. Do not list the materials you used, or the steps taken in a “protocol” format, but incorporate them into the text in paragraph form. Always use past tense and third person (i.e., instead of: ‘I put 10 flies into a climbing apparatus’ write ‘Ten flies were placed in a climbing apparatus’). Use subheadings to clearly separate procedures in this section. Let someone else read this section when you finish writing it and see if the reader could actually do exactly what you did. Please note that citing a lab handout is not acceptable because this is not a published document available to the general public.

Results

This section begins with text; you shouldn’t present tables and/or figures without telling what it is first. The text section is brief and summarizes the content of the figures and tables. Tables and figures should be embedded within the text of the results section after the text where it is mentioned. If you must place the tables/figures on separate pages, they should follow immediately after the page on which they are first mentioned. Tables or figures should be numbered consecutively (Table 1 or Figure 1) and should have a legend (title and explanatory sentences) that explain what is shown. The legend should give enough information so that the table or figure can stand alone, including the p-value and detailed about statistical significance. The results section should not contain an interpretation of the results; this will come in the discussion section.

Use Excel or some other graphing software to produce your graphs, and use Word or Excel to make tables. If you are unclear about which graphing style (scatter plot, bar graph, etc.) to use for different types of data, ask me.

Discussion

This is where you get to be creative and use some independent thought. In the discussion section you interpret your results in terms of how they compare to what you expected, as well as address the broader implications of your results.

You may get unexpected results, or your results may be invalid if the controls in your experiment fail. Is there more than one possible way to explain your data? What are potential sources of error in your results? Give possible scientific/biological reasons for your results, in addition to discussing possible sources of experimental error.

Explain the significance of the results in relation to known scientific literature. When appropriate, propose follow up studies that could refine the results you found. What future experiments could you perform to verify your hypotheses?

Cite any literature you use to support (or refute) your ideas. Your discussion section should end with a conclusions paragraph. The conclusions are not placed in a separate section.

References

Materials that you cite in your paper should be cited according to the CSE Style guide shown below.

**CSE Style Guide**

Citations should be given in “Name Year” format, or in “superscript numerical” format. If citations are in “Name Year” format, references are listed alphabetically at the end of the research paper. If citations are provided as superscript numbers in the text, references are listed at the end of the paper in the order they are cited in the paper.

Examples:

ARTICLE - SCHOLARLY JOURNAL

**References:**

Meise CJ, Johnson DL, Stehlik LL, Manderson J, Shaheen P. 2003. Growth rates of juvenile Winter Flounder under varying environmental conditions. Trans Am Fish Soc 132(2): 225-345.

Do not include retrieval date information or link.

**In-Text:** (Meise et al. 2003)

BOOK

**References:**

McCormac JS, Kennedy G. 2004. Birds of Ohio. Auburn (WA): Lone Pine. 360 p.

**In-Text:** (McCormac and Kennedy 2004)

Excerpt from a sample paper (in Name Year format):

Made naturally, antibiotics are designed to interfere with or kill other microorganisms (Ambile-Cuevas et al. 1995; Levy 1998). Microbes that make the antibiotics have devised ways to protect themselves from their self-manufactured toxins (Ambile-Cuevas et al. 1995). This resistance can also be passed on to other bacteria, even those of different species. When a population of bacteria is exposed to an antibiotic (which occurs, for example, when we take antibiotics), those bacteria NOT resistant to the drug die first. The resistant ones are left behind to produce more resistant bacteria (Ambile-Cuevas et al. 1995; Levy 1998). Some activities contributing to the observed increase in resistant bacteria include the following (in no particular order):

 Not taking antibiotics as prescribed (Ambile-Cuevas et al. 1995; Levy 1998)

 The unnecessary use of antibacterial products (Levy 1998)

 Doctors prescribing antibiotics when they are not needed (Ambile-Cuevas et al. 1995; Levy 1998)

 Mixing antibiotics with livestock feed (Holmberg et al. 1987; Levy 1998)

Resistant bacteria pose an ever-increasing threat. Several strains of the tuberculosis bacterium, resistant to one, two, or even three antibiotics, have been isolated. Some bacteria seem to acquire the DNA of other bacteria, and therefore antibiotic resistance genes, more readily than others (Grady 1996). This has produced particularly pathogenic strains of some food borne bacteria, such as E. coli and Salmonella, which no longer respond to antibiotic treatment (Holmberg et al. 1987). Outbreaks of these infections have made headlines recently. [end of excerpt from text]

**References**

Ambile-Cuevas CF, Cardemas-Garcia M, Ludgar M. 1995. Antibiotic resistance.

American Scientist 83(4): 320-329.

Grady D. 1996. Quick-change pathogens gain on evolutionary edge. Science 274: 1081. Holmberg S, Osterholm M, Sanger K, Cohen M. 1987. Drug-resistant Salmonella from animals fed antimicrobials. New England Journal of Medicine 31: 617-622.

Levy SB. 1998. The challenge of antibiotic resistance. Scientific American 278 (3): 46- 53.

This CSE Style guide was modified from http://library.osu.edu/sites/guides/cbegd.php and

http://www.monroecc.edu/depts/library/cbe.htm.
