# Supplementary material for: A course-based undergraduate research experience examining neurodegeneration in Drosophila melanogaster teaches students to think, communicate, and perform like scientists
Source: PLoS One. 2020 Apr 13;15(4):e0230912. doi: 10.1371/journal.pone.0230912 (PMC7153876; doi:10.1371/journal.pone.0230912)
Supplement: S10 File — Adapted from [33–37]. (DOCX) [file pone.0230912.s011.docx]

**ANALYSIS RUBRIC FOR FINAL LAB REPORTS**

1. **Spelling, Punctuation, Grammar, & Terminology (5% scale) (Communicate)**

Errors in basic spelling/grammar, as well as misuse of scientific terminology (unless the student describes the concept incorrectly, in which case this is a scientific misconception and should be scored in “Conceptual Understanding of Biology Principles” section). If the same mistake is made repeatedly, only count the first instance.

2 = <10 errors

1 = Sporadic errors (10-30)

0 = Many errors (>30) make the paper difficult to read

1. **Format & Style (10% scale) (Communicate)**
2. *Report contains appropriate title (0,1,2)*

2 = Title describes results

1 = “Lab report” or the title of the experiment

0 = No title

1. *Report contains appropriate information in the appropriate sections (with appropriate headings) (0,1,2)*

1 = Appropriate sections and headings, but some information in the wrong section

0 = Missing a section

1. *Report contains properly formatted figures/tables with appropriate numbers and legends (0,1,2)*

2 = Titles with number (e.g, Table 1, Figure 2) and some description

1 = Labeling is incomplete

0 = Figures/tables are embedded within the main paragraphs of the report text with no labeling

1. *Report has a serious tone (no jokes or creative writing, no contractions, no colloquialisms) (0,1,2)*

2 = <10 instances

1 = 10-25 instances

0 = >25 instances

1. *Scientific names are italicized or underlined (0,1,2)*

Includes species names (*e.g., Drosophila melanogaster*) and gene names (1 point for either but not both).

1. **Abstract (10% scale) (Communicate)**
   1. *Single paragraph (0,1,2)*
   2. *Clearly states hypothesis or purpose (0,1,2)*
   3. *Briefly mentions methods (0,1,2)*
   4. *Briefly mentions results (0,1,2)*
   5. *Clearly states the main conclusion(s) (0,1,2)*
2. **Introduction (10% scale) (Communicate: a, b, c & Think: d, e)**
   1. *Provides relevant background/context to explain why this work is of interest (disease relevance, advancing knowledge) (0,1,2)*
   2. *Provides description/background of the model system (0,1,2)*
   3. *Background information is focused on justifying the research question/hypothesis and experiment (not too verbose) (0,1,2)*
   4. *States the research question/hypothesis/purpose (0,1,2)*
   5. *Experimental goals, expected results, methods are summarized (0,1,2)*

1. **Methods (10% scale) (Communicate: a, b, c & Think: d, e)**
   1. *Written with level of detail necessary for a classmate to replicate the experiment (0,1,2)*
   2. *Written in paragraphs and full sentences, not as numbered lists (0,1,2)*
   3. *Appropriate subheadings used to divide the section (0,1,2)*
   4. *States number of replicates used (0,1,2)*

Can be mentioned in figures/tables/results or methods

- 1. *Appropriate rationales given for the methods when necessary (0,1,2)*

Includes mention of control groups and controlled variables, as well as their rationales.

1. **Results (10% scale) (Communicate: c, d, e & Perform: a, b)**
   1. *Reports all appropriate data (comprehensive, relevant, clear, accurate) (0,1,2)*
   2. *Reports all appropriate statistical analyses (comprehensive, relevant, clear, accurate) (0,1,2)*

Includes assumptions of the statistical test (e.g., degrees of freedom, paired or unpaired)

- 1. *Figures, tables, and legends contain sufficient information to be understood outside of the report (0,1,2)*
  2. *States/describes trends or major findings (0,1,2)*

Award 1 point if not included in the Results section but can be found in the Discussion

- 1. *Does not draw conclusions about the data or speculate about anything (such as sources of error) based on the data (0,1,2)*

Limited and simple statements of conclusions are permitted to the extent that it aids in the exposition/narrative of the data but should not include broader speculation*.*

1. **Discussion (10% scale) (Communicate: e & Think: a, b, c, d)**
2. *Draws appropriate conclusions based on the data that are not overly broad (justified, comprehensive, and persuasive) (0,1,2)*
3. *Discusses limitations of results (from design and/or data) and presents how these limitations, or sources of error, moderate the conclusions (0,1,2)*
4. *Provides multiple explanations for results, when appropriate (0,1,2)*

While this is related to subsection b about “sources of error,” here the student should make a separate explicit statement that the sources of error s/he identified provide another possible explanation for the results, rather than simply stating limitations/error.

1. *Proposes future experiments (0,1,2)*

Award credit based on the justification and explanation provided. If report only suggests repeating the same experiment again, award 1 point.

1. *Includes a conclusion paragraph (0,1,2)*

A single conclusion sentence earns 1 point.

1. **Literature Cited (5% scale) (Perform)**
   1. *Sufficient and appropriate sources cited (0,1,2)*

2 = Report uses more than five unique resources and includes at least one scientific paper

1 = At least two web sources found on their own (i.e., not the textbook, lecture notes, or the citation from the handouts provided) or at least one primary research article

- 1. *Sources are cited appropriately in the text (0,1,2)*

*c. Literature Cited/References section is present and formatted consistently (0,1,2)*

Award 1 point if the references section is present but lacking formatting*.*

1. **Conceptual Understanding of Biology Principles (15% scale)**

**2018 Reports:**

1. *Accurately describes GAL4-UAS system (0,1,2)*

Includes that GAL4 is a transcription factor and activates transcription of UAS transgene downstream

1. *Accurately describes how RNAi works (distinguishing it from mutation) (0,1,2)*

Includes that RNAi degrades mRNA, preventing/reducing gene expression/protein function

1. *Describes that the genetic manipulation is occurring in subsets of neurons (as directed by GAL4 driver) (0,1,2)*
2. *Any other miscellaneous statement that is inaccurate or reflects a misconception (-2,-1,0)*

Any other inaccurate statement not captured by the above concepts. Count each misconception once.

-2 = A major misconception or multiple minor misconceptions

-1 = One or two minor misconceptions

**2017 Reports:**

- 1. *Genes are on chromosomes (0,1,2)*
  2. *Each gene has a distinct and fixed locus (0,1,2)*
  3. *A gene’s map position can be determined based on recombination frequencies (0,1,2)*
  4. *Any other miscellaneous statement that is inaccurate or reflects a misconception (-2,-1,0)*

1. **Conceptual Understanding of Scientific Method Principles (15% scale)**
   1. *Hypothesis/purpose of experiment is stated clearly (0,1,2)*
2. *Biological rationale for hypothesis (0,1,2)*
3. *Identifies independent and dependent variables (0,1,2)*

Does not require using the terms independent and dependent variables, per se. A description of what was manipulated and what was measured is sufficient.

1. *Describes how the dependent variable is going to be measured (and how the independent variable will be manipulated) (0,1,2)*
2. *Demonstrates an understanding that all other variables need to be held as constant as possible (0,1,2)*
3. *Identifies experimental and control groups (0,1,2)*
4. *Demonstrates an understanding of the importance of repetition and sample size (0,1,2)*

2 = Describes repetition or sample size as a source of error or proposed future experiment (indicating its importance

1 = Simply mentions n in methods/results without description of its importance

0 = No mention of n anywhere (including in reporting data)

1. *States conclusions that are appropriately narrow (indicates awareness of multiple explanations or possible sources of error) (0,1,2)*
2. *States that the evidence provides support for hypothesis (or not), not that it “proves” (0,1,2)*

Score this by use of keyword “proves”. Conclusion statements should be framed as “evidence” that “supports” a hypothesis. Award 1 point if the student contradicts him-/herself or offers inconsistent understanding of this key scientific concept.

1. *Describes future experiments to distinguish multiple explanations (0,1,2)*

2 = Includes any reasonably well-justified future experiment suggestion

1 = Repeating the experiment without justification

0 = No future experiments are suggested
